# Microwave-Assisted Incorporation of AgNP into Chitosan–Alginate Hydrogels for Antimicrobial Applications

**DOI:** 10.3390/jfb14040199

**Published:** 2023-04-04

**Authors:** Takuma Oe, Duangkamol Dechojarassri, Sachiro Kakinoki, Hideya Kawasaki, Tetsuya Furuike, Hiroshi Tamura

**Affiliations:** 1 Faculty of Chemistry, Materials and Bioengineering, Kansai University, Osaka 564-8680, Japan; 2 Organization for Research and Development of Innovative Science and Technology (ORDIST), Kansai University, 3-3-35 Yamate-cho, Suita, Osaka 564-8680, Japan

**Keywords:** chitosan, alginate, silver nanoparticles, microwave, hydrogel, antimicrobial applications

## Abstract

Herein, improving the antibacterial activity of a hydrogel system of sodium alginate (SA) and basic chitosan (CS) using sodium hydrogen carbonate by adding AgNPs was investigated. SA-coated AgNPs produced by ascorbic acid or microwave heating were evaluated for their antimicrobial activity. Unlike ascorbic acid, the microwave-assisted method produced uniform and stable SA-AgNPs with an optimal reaction time of 8 min. Transmission electron microscopy (TEM) confirmed the formation of SA-AgNPs with an average particle size of 9 ± 2 nm. Moreover, UV-vis spectroscopy confirmed the optimal conditions for SA-AgNP synthesis (0.5% SA, 50 mM AgNO_3_, and pH 9 at 80 °C). Fourier transform infrared (FTIR) spectroscopy confirmed that the –COO^−^ group of SA electrostatically interacted with either the Ag^+^ or –NH_3_^+^ of CS. Adding glucono-δ-lactone (GDL) to the mixture of SA-AgNPs/CS resulted in a low pH (below the p*K*a of CS). An SA-AgNPs/CS gel was formed successfully and retained its shape. This hydrogel exhibited 25 ± 2 mm and 21 ± 1 mm inhibition zones against *E. coli* and *B. subtilis* and showed low cytotoxicity. Additionally, the SA-AgNP/CS gel showed higher mechanical strength than SA/CS gels, possibly due to the higher crosslink density. In this work, a novel antibacterial hydrogel system was synthesized via 8 min of microwave heating.

## 1. Introduction

The subject of wound dressing has long been investigated in the medical field to promote cell growth and inhibit the proliferation of bacteria in the wound. For the former purpose, the structures should be interconnected, porous, and have a high surface-to-volume ratio, such as electrospun nanofibers [1,2]. For the latter, the materials should possess antibacterial activity, such as materials with specific functional groups or composite materials whose fillers have antibacterial properties. Therefore, electrospun nanofibrous structures made with antibacterial nanoparticles would be the best choice [3,4]. However, despite its availability, electrospinning still takes a very long time to form a film for practical use.

Alginate (Alg) is a linear unbranched polysaccharide comprising (1–4)-linked β-D-mannuronic acid and its epimer α-D-guluronic acid. Alg-based materials have been reported to be suitable for wound dressings [5,6] owing to their good antimicrobial activity, gelling ability, biocompatibility, and hydrophilicity. Another biopolymer that has been applied in medical applications is chitosan (CS) because of its excellent properties, such as biodegradability, biocompatibility, low cytotoxicity, and antimicrobial activity. Recently, research into CS and Alg-based hydrogels for wound-healing applications has gained more attention because both hydrogels can prevent the loss of water from the wound. This leads to a prolonged moist environment that minimizes bacterial infection in the wound while also absorbing excess wound exudate [7,8].

In the preparation of hydrogels, the appropriate solvent has to be chosen. It is commonly known that CS and Alg can be dissolved in an acid solution and pure water, respectively. If the acidic CS solution is mixed with an aqueous solution of Alg, the highly protonated CS (cation) chain has been reported to rapidly interact with an anionic chain of Alg and form a precipitate instead of a uniform gel [9]. Therefore, the conditions for forming a hydrogel from CS and Alg has to be modified. Although CS cannot usually be dissolved in a basic solution, it is soluble in sodium hydrogen carbonate (NaHCO_3_). Recently, uniform gels prepared from the basic CS (cation) and sodium alginate (SA, anion) were proposed by our group [10].

NaHCO_3_ was first added to a solution of CS in acetic acid to prepare a basic CS solution. This basic condition assured a lower degree of protonation in the CS chains. Subsequently, SA and glucono-δ-lactone (GDL) were added to the basic CS solution. The pH of the mixture was then gradually decreased until its pH was lower than the p*K*a of CS (~6.5). Eventually, the uniform gel turned into a SA/CS hydrogel [10].

The successful use of nanomaterials in biological applications requires the evaluation of various parameters such as antibacterial activity, cytotoxicity, and mechanical strength, to name a few. To boost the antibacterial activity, silver nanoparticles (AgNPs), widely acknowledged for their excellent antimicrobial activity [11,12,13,14,15,16,17,18], could be incorporated into the working materials to yield an effective nanocomposite. The most common method to synthesize AgNPs is a reduction reaction, in which reducing agents in the presence of stabilizers are utilized [19,20,21,22], but some of the chemicals used in this reaction are highly toxic to both the environment and living organisms. Hence, green synthesis of AgNPs, using non-toxic and environmentally friendly substances has been extensively investigated [23,24,25,26,27]. During the synthesis, a mixture of all the reactants needs to be heated for the reduction to occur. Microwave heating, an attractive heating alternative, has been reported to yield uniform AgNPs because of its ability to significantly accelerate the reaction rate in a short time, and prevents aggregation during synthesis by forming uniform nucleation sites [28,29]. To control the sizes of nanoparticles and reduce their cytotoxicity, coating nanoparticles with different polysaccharides was found to be effective [30,31,32]. For example, Chen et al. found that PVA/SA/carboxymethyl chitosan (PVA/SA/CMCS) hydrogels containing SA-AgNPs (synthesized using 0.05% AgNO_3_ and 0.2% SA at 90 °C for 12 h) demonstrated 21 and 20 mm inhibition zones against *E. coli* and *S. aureus*, respectively. In addition, PVA/SA/CMCS hydrogels with SA-AgNPs showed low cytotoxicity with a cell viability of 80% [33].

This study focused on enhancing the antibacterial activity of SA/CS gel prepared via our method [10] by incorporating AgNPs (synthesized with microwave heating) to shorten the reaction time. In addition, AgNPs were coated with SA (SA-AgNPs) to reduce the cytotoxicity of AgNPs. In this work, a SA-AgNPs/CS gel was successfully synthesized. It was suggested that the –COO^−^ group of SA could electrostatically interact with Ag^+^ and help control the size of SA-AgNPs during particle growth. When CS was added later, the –COO^−^ group of the SA that was already coated on the SA-AgNPs electrostatically interacted with the protonated –NH_3_^+^ group of CS. As a result, the SA-AgNPs/CS colloid was formed into a hydrogel by adding GDL. This new system has not been investigated before. The obtained hydrogel possessed good properties, such as retaining its shape (thus yielding a slow Ag^+^ release), exhibiting good water absorption, and having good stability. The antibacterial activity was boosted, and the mechanical strength of the SA-AgNPs/CS gels was superior to SA/CA gels, possibly due to the reinforcing effect of the SA-AgNPs homogeneously dispersed in the SA/CA matrix, which led to the higher crosslink density of the whole nanocomposite.

## 2. Materials and Methods

### 2.1. Materials

Sodium alginate (SA, grade I-S, M/G = 1.0, viscosity of 1% SA = 1.031 mPa·s) and chitosan (CS, FL-80, Mw = 5.3 × 10^4^, DAC: 85.6) were supplied by Koyo Chem. Co., Ltd. (Osaka, Japan) and Kimica Corporation (Tokyo, Japan). Silver nitrate (AgNO_3_), citric acid, sodium hydrogen carbonate (NaHCO_3_), sodium hydroxide (NaOH), acetic acid, and L-glutamic acid were purchased from Wako Pure Chemical Industries Ltd. (Osaka, Japan). Glucono-δ-lactone (GDL) was purchased from Tokyo Chemical Industry Co., Ltd. (Tokyo, Japan), while LB agar and LB broth were purchased from Becton, Dickinson and Company (New York, USA). Eagle’s MEM (EMEM) was purchased from Nissui Pharmaceutical Co., Ltd., Ibaraki, Japan. Fetal bovine serum (FBS) was purchased from Biowest Company (Lakewood Ranch, FL, USA). A high-density polyethylene (HDPE) film and a polyurethane (PU) film with 0.25% zinc dibutyldithiocarbamate (ZDBC) were purchased from the Food and Drug Safety Center (Kanagawa, Japan). BALB-3T3 clone A31 cells (mouse fibroblasts, resource No. RCB0005, lot No. 16, Passage: 3) were purchased from Riken BRC Cell. Bank, Japan. All chemicals were used without any treatment.

### 2.2. Preparation of SA-AgNPs

#### 2.2.1. Synthesis without Microwave Irradiation

The SA-AgNP solution was prepared by adding 1 mL of a AgNO_3_ solution (50 mM) to 10 mL of 0.5% SA and stirred at 25 °C for 1 min. Subsequently, 1 mL of ascorbic acid (50 mM) was added, followed by the addition of a NaOH solution (0.1 M) to adjust the pH to 9. A final AgNO_3_ concentration of 0.004 mM/mL was obtained. The SA-AgNP solution was produced after a synthesis time of 1 min, and its absorbance was measured using a UV–vis spectrophotometer (UV–2910, HITACHI, Tokyo, Japan) with a scanning wavelength range of 300–600 nm.

#### 2.2.2. Synthesis by Microwave Irradiation

SA-AgNPs were synthesized by completely dissolving 0.05 g of SA in 10 mL of deionized (DI) water, and the pH value of the solution was adjusted within the range of 5–11 by adding a NaOH solution (0.1 M). Subsequently, 1 mL of a AgNO_3_ solution was added, and the reaction mixture, held in a 100 mL glass vessel, was inserted into a microwave device (Discover Labmate, CEM Corporation, North Carolina, USA) with a magnetron frequency of 2450 MHz, a power output of 300 W, a power output per reaction volume of 3 W/mL, and a processing pressure of 1.013 bar to reduce the Ag^+^ and form SA-AgNPs. Afterwards, the mixture was cooled in an ice bath to stop the reaction. The concentrations of the initial SA and AgNO_3_ solution were in the ranges of 0.1–2.0% and 10–500 mM, respectively, while the reaction temperature and time were in the ranges of 60–90 °C and 1–15 min, respectively. The final concentration of the SA-AgNPs was detected using ICP–AES (ICP-7510, Shimadzu, Kyoto, Japan).

### 2.3. Preparation of the SA-AgNPs/CS gel

The SA-AgNPs/CS gel was prepared according to the method described by [10]. Initially, pure SA was added to the SA-AgNP solution (synthesized according to Section 2.2.1 and Section 2.2.2) and stirred overnight to form a 2.0% SA-AgNP solution. Subsequently, 191.0 mg of CS was mixed with 1 mL of DI water and 102.2 µL of an acetic acid solution (1 M) at a molar ratio a CS monomer unit:acetic acid of 1:1. Sodium bicarbonate powder (0.382 g) was then added to the CS solution and stirred vigorously to stabilize the basic CS solution. Next, 1 mL of the 2.0% SA-AgNP solution was mixed with 1 mL of the basic CS solution and stirred until homogeneous, followed by the addition of a GDL solution (1 M). Subsequently, this solution formed the SA-AgNPs/CS gel after standing at room temperature in the dark for 1 h.

### 2.4. Characterization of the Synthesized AgNPs, SA-AgNPs, and SA-AgNPs/CS Gel

The formation of AgNP was confirmed by measuring the color change of the solution using a UV-vis spectrophotometer. Transmission electron microscopy (TEM) micrographs of the SA-AgNPs were recorded by a transmission electron microscope (TEM, JEM-1400Flash, JEOL, Tokyo, Japan) at an acceleration voltage of 100 kV. Fourier transform infrared (FTIR) spectroscopy (FT/IR-6300, JASCO, Tokyo, Japan) with the KBr method was used to record FTIR spectra of the freeze-dried samples. Field emission scanning electron spectroscopy (FE-SEM, JSM6700, JEOL, Japan) was used to analyze the structural changes of the gels with different CS:SA values.

### 2.5. Compressive Strength, Crosslink Density, and SEM Images of SA-AgNPs Gel

A universal testing machine (EZ test series, Shimadzu, Japan) was used for compressive-strength tests. Hydrogels formed using different SA-AgNPs/CS compositions (with the AgNO_3_ concentration and CS:SA in the ranges of 10–50 mM and 0.5–1:1, respectively) were used for compressive-strength tests. The crosslink densities of the samples were calculated from the Young’s modulus evaluated using Equations (1) and (2) [34]:

Crosslink densities (mol/m^3^) = Young’s modulus (N/m^2^)/*Φ*^1/3^ × *R* × *T*, (1)

*Φ* = weight of the gel in the dried state (g)/weight of the gel in the wet state (g), (2)

where *R* is 8.314 (J/mol ∙ K) and *T* is the measured temperature (K). The SA-AgNPs/CS gels were freeze-dried and evaporated using a platinum evaporation apparatus. The average value and the standard deviation were calculated from 5-time measurements and reported.

### 2.6. Water-Resistance Test of the SA-AgNPs/CS Gel

The swelling ratio of the hydrogels in DI water indicated their water resistance. Firstly, excess water at the hydrogel surface was gently wiped off, followed by weighing. Subsequently, the hydrogel was immersed in 100 mL of DI water for three days; the swollen-state hydrogel was weighed in 24-h intervals. Finally, the samples were freeze-dried and weighed in their dried state. The swelling ratio was determined using Equation (3):

Swelling ratio = *W*_w_/*W*_d_, (3)

where *W*_w_ (g) and *W*_d_ (g) are the gel weights in the wet and dried states, respectively. The average value and the standard deviation were calculated from 5-time measurements and reported.

The SA-AgNPs/CS hydrogels were then immersed in 100 mL of DI water for seven days to measure the release of Ag^+^. Every 24 h, pure DI water was replaced. The amount of Ag^+^ in 5 mL of the immersed solution was detected using inductively coupled plasma atomic emission spectroscopy (ICP–AES, ICP-7510, Shimadzu, Japan).

### 2.7. Antibacterial Test

The disc diffusion method was used to analyze the antibacterial activity of the SA-AgNPs/CS gels with AgNO_3_ concentrations ranging from 10–50 mM. *Escherichia coli* (*E. coli*) and *Bacillus subtilis* (*B. subtilis*) were used as model test strains for gram-negative and gram-positive bacteria. Firstly, *E. coli* or *B. subtilis* were added to Luria-Bertani (LB) broth solution and pre-cultured overnight with a shaking speed of 180 rpm at 37 °C. Then, the pre-cultured bacterial solution was diluted to 1.0 × 10^6^ CFU/mL. LB agar (0.8 g) was added to 20 mL of DI water and sterilized in an autoclave at 121 °C for 15 min. The agar medium was then transferred to a sterile petri dish. Finally, after solidification, 0.1 mL of pre-cultured bacterial solution was spread on the medium surface and incubated overnight at 37 °C. The zone of inhibition for each sample was recorded using a digital camera and analyzed. The average value and the standard deviation were calculated from 3-time measurements and reported.

### 2.8. Cytotoxicity Test

#### 2.8.1. Cell Propagation for Cytotoxicity Test

BALB-3T3 clone A31 cells (mouse fibroblasts) were used as the cell model to analyze the cytotoxicity of the SA-AgNPs/CS and SA/CS gels. Before experimentation, BALB-3T3 clone A31 cells (mouse fibroblasts) were propagated. Firstly, 4.7 g of EMEM and 0.75 g of NaHCO_3_ were dissolved in 440 mL of DI water. After that, 0.146 g of L-glutamic acid, 50 mL of FBS solution, and 10 mL of DI water were added to the solution. The obtained solution was sterilized and used as the new medium. Then, 1 mL of BALB-3T3 clone A31 cells was added to 10 mL of the sterilized medium and incubated at 37 °C for 24 h. Finally, the old medium was removed, and replaced with fresh medium, and incubated for 72 h. Meanwhile, the samples were lyophilized for two days and sterilized using ethylene oxide gas (EOG). The obtained solution was sterilized and is also referred to in this study as “medium”.

#### 2.8.2. Cytotoxicity Test

Firstly, the SA-AgNPs/CS gel and SA/CS gel were freeze-dried for 48 h, and 0.1 g of the freeze-dried sample was added to 5 mL of medium and incubated for 72 h. We will further refer to this filtrate as “extract”. The medium was removed from the 96-well plates containing the BALB-3T3 clone A31 cells with a concentration of 1 × 10^3^ cells/well (at 3 log phase). Then, 100 μL of the extract was added to the 96-well plates and incubated at 37 °C for 72 h. The SA/CS gel extract (a blank), the HDPE film extract (a negative control), and the PU film with 0.25% ZDBC extract (a positive control) were tested under the same conditions. Subsequently, the cells were treated with a new extract and incubated for another 72 h. Finally, after incubation, the cell counts were determined using Cell Counting Kit 8 (Dojindo, Kumamoto, Japan). The cell viability was determined using Equation (4):

Cell viability (%) = *Abs*_sample_/*Abs*_blank_ × 100, (4)

where *Abs*_sample_ and *Abs*_blank_ are the extracts of the SA-AgNPs/CS gel (or negative control or positive control) and the extract of the SA/CS gel, respectively. The average value and the standard deviation were calculated from 3-time measurements and reported.

## 3. Results and Discussion

### 3.1. Reaction Mechanism of AgNP Formation

**Figure 1:**
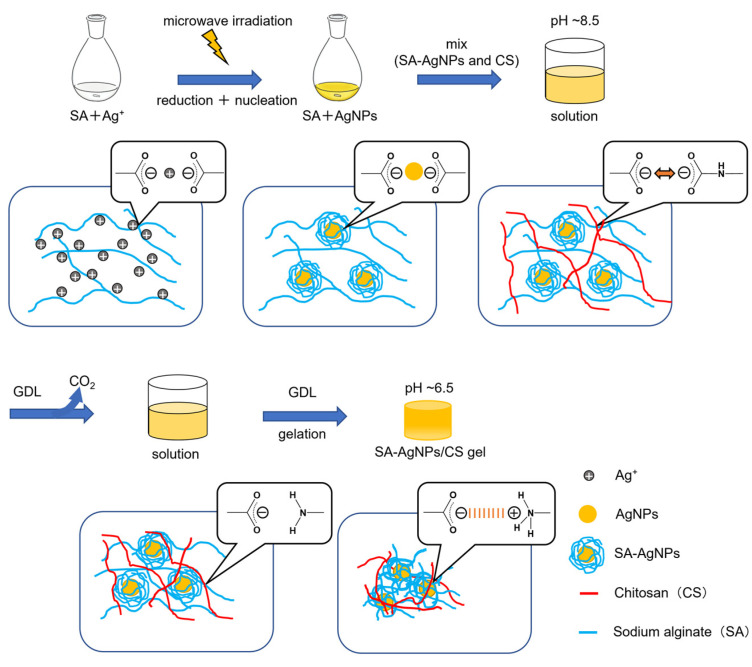
Schematic illustration of the SA-AgNPs/CS gel fabrication.

Forming uniform SA-AgNPs requires a reducing agent for Ag^+^ reduction and a stabilizer to prevent AgNP aggregation. Herein, SA was used as the reducing agent and the stabilizer when forming SA-AgNPs. The overall synthetic procedure for this experiment is shown in Figure 1.

According to previous reports, the mechanism of SA-AgNP formation can be divided into two stages [35]. The first stage involves the reduction of Ag^+^ to AgNPs by the available reduction groups. As nucleation centers in the second stage, the resultant AgNPs are said to catalyze the reduction of the remaining Ag^+^ in the bulk solution and adsorb Ag on their surface. This process is repeated to form large particles. SA stabilizes AgNPs by its carboxy (–COO^−^) groups, preventing further aggregation; (the electrostatic repulsion by the –COO^−^ groups of Alg and steric hindrance by the Alg chains prevent aggregation) [28]. The formation of the gel could be completed after adding GDL. The pH of the prepared SA-AgNPs mixed with basic CS solution gradually lowers. The resulting SA-AgNPs/CS gel is formed successfully below the p*K*a of CS (~6.5) and retains its shape.

### 3.2. Confirmation of SA-AgNP Synthesis

#### 3.2.1. Synthesis without Microwave Irradiation

**Figure 2:**
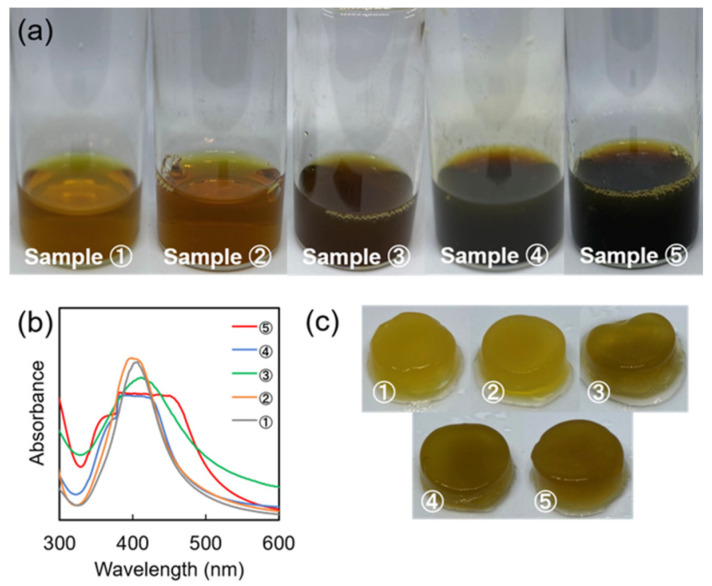
SA-AgNPs synthesized without microwave irradiation. (**a**) Photographs of the SA-AgNP suspensions. (**b**) UV-vis spectra of the AgNPs. (**c**) Hydrogel fabricated using the SA-AgNP suspensions.

UV-vis spectroscopy, a simple and sensitive method, was used to characterize the SA-AgNPs by their excitation surface plasmons. Theoretical studies on the size dependence of metallic spheres on UV-visible absorption have indicated that the surface plasmon resonance band shows a red shift with increasing particle size and a blue shift with decreasing particle size [36]. Colloidal silver aggregation causes a decrease in the main peak’s intensity, with the appearance of an additional blue peak on the long wavelength side [37]. After the reaction without microwave irradiation, the color of the SA-AgNPs changed. Figure 2a–c show the SA-AgNP suspensions, their UV absorbance values, and the hydrogels fabricated using the suspensions. After the experiment had been five times, the SA-AgNP suspensions’ color, UV spectra, and hydrogels were unique each time. Particularly, the UV spectra of the solution, with a peak around 400 nm representing AgNPs, was observed only in experiments No. 1 (sample ①) and No. 2 (sample ②), whereas a peak at 410 nm corresponding to larger AgNPs was observed in experiment No. 3 (sample ③). Experiments No. 4 (sample ④) and 5 (sample ⑤) did not show any clear peaks. This could be due to the small amount of SA used, which was insufficient to simultaneously function as a reducing agent and a stabilizer. Although ascorbic acid was added to the system, the SA did not wholly coat the AgNPs in the system during the reaction. As this synthesis (without microwave irradiation) was not reproducible, SA-AgNPs were synthesized with microwave irradiation for the remaining experiments.

#### 3.2.2. Synthesis Using Microwave Irradiation

**Figure 3:**
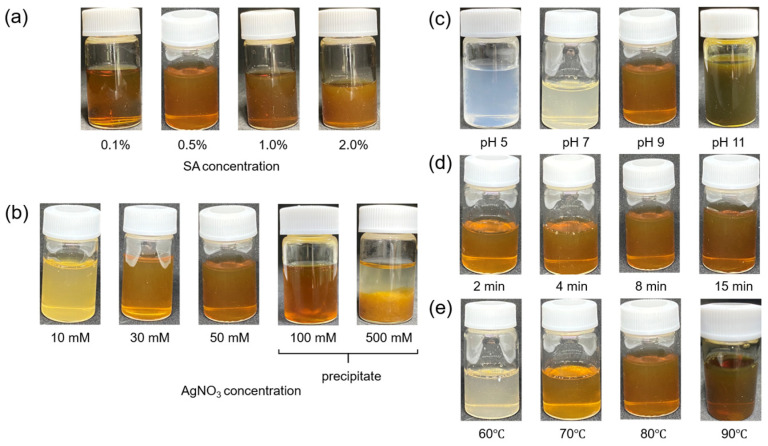
Colors exhibited by the SA-AgNP suspensions with (**a**) 50 mM AgNO_3_ at pH 9 and 80 °C, after 8 min of reaction with various SA concentrations; (**b**) 0.5% SA at pH 9 and 80 °C, after 8 min of reaction with various AgNO_3_ concentrations; (**c**) 0.5% SA and 50 mM AgNO_3_ at 80 °C and different pH values after 8 min of reaction; (**d**) 0.5% SA and 50 mM AgNO_3_ at pH 9 and 80 °C, with different irradiation times; and (**e**) 0.5% SA and 50 mM AgNO_3_ at pH 9 and different temperatures, after 8 min of reaction.

**Figure 4:**
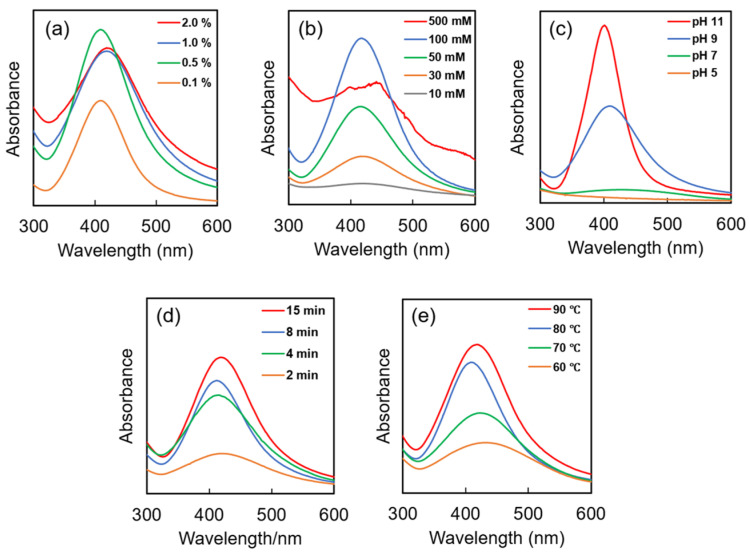
UV-vis spectra of AgNPs fabricated using (**a**) 50 mM AgNO_3_ at pH 9 and 80 °C, after 8 min of reaction with various SA concentrations; (**b**) 0.5% SA at pH 9 and 80 °C, after 8 min of reaction with different AgNO_3_ concentrations; (**c**) 0.5% SA and 50 mM AgNO_3_ at 80 °C with various pH values, after 8 min of reaction; (**d**) 0.5% SA and 50 mM AgNO_3_ at pH 9, 80 °C, and different irradiation times; and (**e**) 0.5% SA and 50 mM AgNO_3_ at pH 9 and different temperatures, after 8 min of reaction.

After microwave irradiation, the SA solution with AgNO_3_ changed from colorless to light yellow to reddish-brown, according to the conditions of synthesis (Figure 3a–e). The change in the color of the solution indicated the formation of SA-AgNPs. Figure 4a shows the UV-vis spectrum of SA-AgNPs synthesized with different SA concentrations (0.1–2.0%) and a fixed AgNO_3_ concentration (50 mM) at pH 9, with microwave irradiation at 80 °C for 8 min. The intensity of absorbance increased with an increase in the SA concentration from 0.1 to 0.5%, indicating an increase in the formation of SA-AgNPs, whereas it decreased when the SA concentration was increased to 1% and 2%, indicating a smaller amount of SA-AgNPs. Additionally, when 1% and 2% of SA was used, the absorption peak exhibited a red shift to the longer wavelength side, indicating the formation of SA-AgNPs with a larger particle size [38]. Microwave radiation was unable to optically vibrate the SA molecules at high concentrations, thereby causing an inefficient acceleration of the SA molecule–AgNP interaction. Thus, the optimal SA concentration for microwave synthesis is 0.5%.

Subsequently, the formation of SA-AgNPs using 0.5% SA at different concentrations of AgNO_3_ (10–500 mM) was evaluated. As shown in Figure 4b, the formation of SA-AgNPs increased with an increase in the concentration of AgNO_3_ up to 50 mM, while precipitation was observed beyond 100 mM (Figure 3b); significant precipitation occurred, facilitating the separation of the water layer from the precipitate layer. In addition, SA produces aggregates with metal ions [39]. This experiment confirmed the aggregation of SA at high concentrations of Ag^+^; therefore, the optimal concentration of AgNO_3_ for microwave synthesis was 50 mM, with the ABS peak of 410 nm corresponding to AgNPs.

High-pH solutions exhibited strong turbidity and a blue-shifted peak at pH 11 (Figure 4c); moreover, the absorption peak intensity increased with an increase in the pH. These phenomena could be attributed to the increased electrostatic exposure of the carboxy groups of SA due to the higher pH of the solution [28]. The blue shift indicated a decrease in particle size. It has been found that large amounts of AgNP with a small particle size exhibit high cytotoxicity [40].

At pH values less than 7, the solution did not exhibit a yellow color; however, it was reddish-brown at pH 9. As mentioned previously and confirmed by the UV-vis absorbance measurements, the color change indicated the formation of AgNP. In high-pH solutions, Ag^+^ reacts with OH^−^ to form a silver complex that is subsequently reduced to AgNPs [41,42].

SA is a weak reducing agent, and the general external heating method requires a longer reaction time than microwave heating. Consequently, the method is associated with the agglomeration of AgNP due to the thermal decomposition of SA by prolonged heating, which is a severe limitation that requires resolution. Here, the rapid and uniform heating profile facilitated a microwave reaction with a short reaction time. As shown in Figure 4d, the peak intensity of absorption increased with the reaction time. The peak showed a larger red shift to the longer wavelength side after 15 min than that exhibited after 8 min, indicating an increase in the size of AgNP particles [30]. The optimal reaction time in this study was 8 min, consistent with the results of Zhao et al. [28]. Moreover, according to a report by Peng et al. on the synthesis of AgNPs using bamboo hemicellulose via microwaves, forming AgNPs with the reducing agent glucose requires a reaction time of more than 2 min [43].

The effect of temperature on the morphology of AgNPs has been reported in previous studies [44]. As shown in Figure 4e, the formation of AgNP increased with the reaction temperature, and a slightly red-shifted peak was observed at a reaction temperature of 90 °C. This could be attributed to a disruption of the SA coating layer due to the agglomeration of AgNP because of the enhanced thermally induced molecular motion after the increase in the temperature.

**Figure 5:**
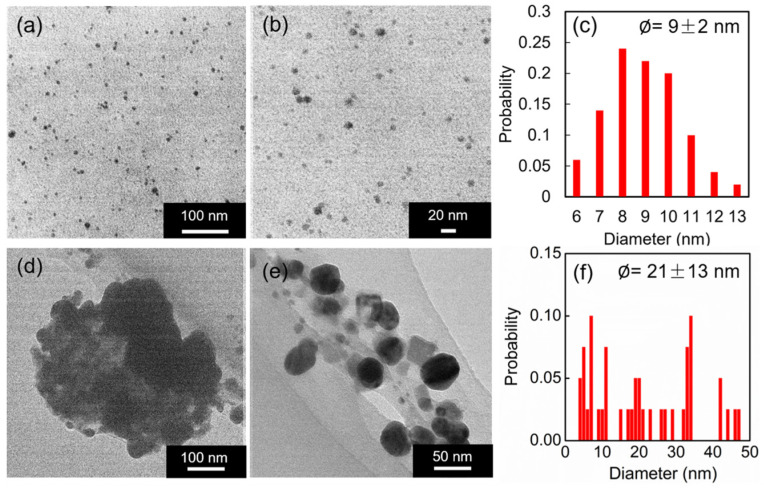
TEM images of SA-AgNPs fabricated using 0.5% SA and 50 mM AgNO_3_ at pH 9 and 80 °C, after 8 min of reaction: (**a**) with a scale bar of 100 nm and (**b**) with a scale bar of 20 nm. (**c**) The size distribution of the SA-AgNPs. TEM images of the AgNPs fabricated without the SA coating using 50 mM AgNO_3_ at pH 9 and 80 °C, after 8 min of reaction: (**d**) with a scale bar of 100 nm and (**e**) with a scale bar of 50 nm. (**f**) The size distribution of the AgNPs without the SA coating.

Figure 5 shows the TEM images of the SA-AgNP sample synthesized with 0.5% SA and 50 mM AgNO_3_ at pH 9 and 80 °C, after a reaction time of 8 min. A uniform dispersion of spherical SA-AgNPs was observed, with an average particle size of 9 ± 2 nm, indicating the significant role of SA as a stabilizer. Contrariwise, AgNPs without the SA coating exhibited self-agglomeration with larger diameters of 21 nm and a wider size dispersity of 13 nm. According to a report by Agnihotri et al., AgNPs with an average particle size of 5–100 nm exhibit antimicrobial properties against various bacteria, and those with particle sizes below 10 nm exhibit the best antibacterial activity against *E. coli*. [19]. Moreover, the spherical shape of AgNPs gives higher bactericidal efficacy against *E. coli.* than rod-shaped AgNPs [45]. The SA exhibited reducing and stabilizing properties; thus, the SA coating facilitated the production of high-stability uniform SA-AgNPs with an average diameter of 10 nm. The SA-AgNPs showed high antimicrobial activity [28]; thus, the SA-AgNPs synthesized here were expected to exhibit good antimicrobial activity.

### 3.3. Preparation of the SA-AgNPs/CS Gel

**Figure 6:**
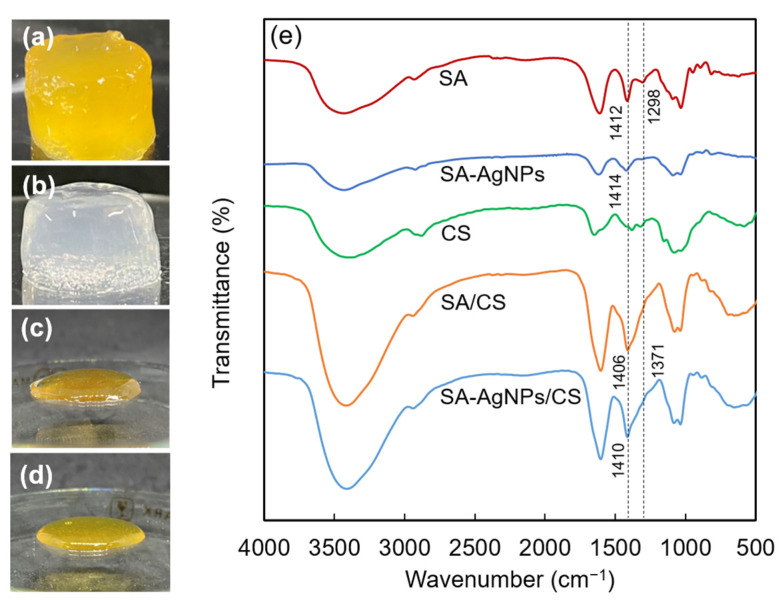
Digital photographs of (**a**) the SA-AgNPs/CS, (**b**) the SA/CS gel, (**c**) the mixture of SA-AgNPs and CS without NaHCO_3_ and GDL, and (**d**) the mixture of SA-AgNPs and CS with the addition of NaHCO_3_ without GDL. (**e**) The FTIR spectra of SA, the SA-AgNPs, CS, the SA/CS gel, and the SA-AgNPs/CS gel.

Firstly, SA-AgNPs were synthesized by completely dissolving 0.05 g of SA in 10 mL of DI water to achieve an SA concentration of 0.5%. Secondly, 1 mL of a 50 mM AgNO_3_ solution was added, and the pH value of the solution was adjusted to 9 by adding a 0.1 M NaOH solution. The reaction mixture was then inserted into a microwave device at 80 °C for 8 min to reduce the Ag^+^ and form SA-AgNPs. Afterwards, the mixture was cooled in an ice bath to stop the reaction. During the reaction, the –COO^−^ group of SA, acting as the reducing and stabilizing agent, interacted with the Ag^+^, and then the Ag^+^ was changed to Ag^0^ by microwave irradiation. After the SA-AgNPs had been obtained, CS was added and mixed. Finally, GDL was added according to the previous method [10]. The pH of the system gradually decreased to 6.5, which is the p*K*a value of CS. At this pH, NH_2_ is protonated to –NH_3_^+^, and the –NH_3_^+^ interacts with the –COO^−^ of SA. The SA-AgNPs/CS gel was obtained as explained in Figure 1. The preparation of the SA-AgNPs/CS gels was confirmed by FTIR, with color changes due to plasmon absorption by the AgNPs. The gel containing SA-AgNP retained its shape and remained bright yellow, unlike the SA/CS gel without SA-AgNPs, as shown in Figure 6a,b. Additionally, the mixture of SA-AgNPs and CS without the addition of NaHCO_3_ and GDL did not form a gel, as shown in Figure 6c. The mixture of SA-AgNPs and CS with the addition of NaHCO_3_ and without GDL also did not form a gel, as shown in Figure 6d. The FTIR spectra of SA, CS, the SA-AgNP solution, the SA/CS gel, and the SA-AgNPs/CS gel are shown in Figure 6e. The peaks at 1298 and 1412 cm^−1^ corresponded to the –COO^−^ groups of the SA molecules. These peaks overlapped to form a single peak at 1414 cm^−1^ in the spectrum of SA-AgNPs; the higher wavelength shift of the peak indicated an increase in the binding force between AgNPs and the –COO^−^ groups. The band at 1371–1406 cm^−1^ in the SA/CS gel shifted to the higher wavelength side in the SA-AgNPs/CS gel (to 1410 cm^−1^), indicating that the AgNPs interacted with the –COO^−^ group of SA, consistent with the stabilization mechanism of SA-AgNP [46].

### 3.4. Mechanical Properties and SEM Images of the SA-AgNPs/CS Gels

**Figure 7:**
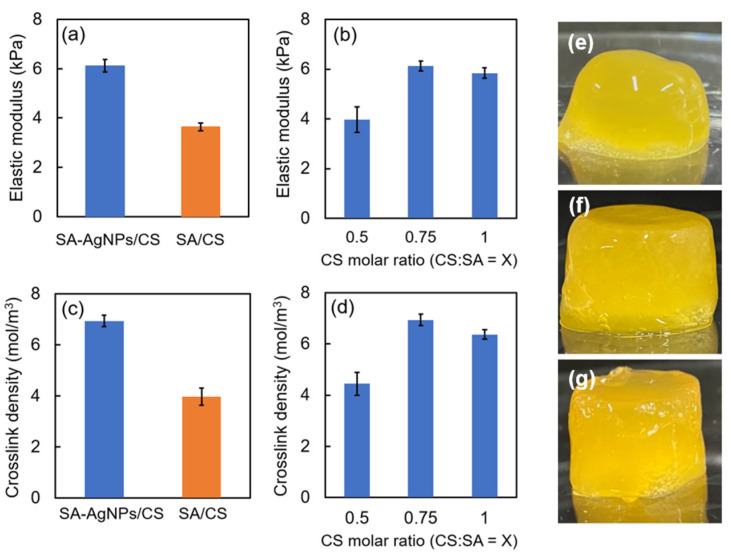
Young’s modulus of (**a**) the SA-AgNPs/CS and SA/CS gels (CS:SA = 0.75:1) and (**b**) the SA-AgNPs/CS gel with different molar ratios of CS:SA. Crosslinking densities of (**c**) the SA-AgNPs/CS and SA/CS gels (CS:SA = 0.75:1) and (**d**) the SA-AgNPs/CS gel with different molar ratios of CS:SA. Digital photographs of the SA-AgNPs/CS gel with different molar ratios of CS:SA: (**e**) CS:SA = 0.5:1, (**f**) CS:SA = 0.75:1, and (**g**) CS:SA = 1:1.

High antibacterial activity and low cytotoxicity are required for biomedical applications of a material, in addition to the high mechanical strength of the material. In our previous work, the ratio of CS:SA in the SA/CS gel was fixed at 1:1 [10]. Here, the enhancements in the strength of the SA/CS gel by varying the ratio of CS to SA were investigated. The strength of the SA-AgNPs/CS gels is shown in Figure 7a,b.

The SA-AgNPs/CS gel exhibited a higher Young’s modulus than the SA/CS gel without SA-AgNPs. When the ratio of CS:SA in the SA-AgNPs/CS gels was varied (0.5, 0.75, and 1), the gel with a CS:SA value of 0.75 exhibited the most prominent Young’s modulus, similar to the trend exhibited by the crosslinking densities of the gels (Figure 7c,d). Furthermore, digital photographs of the SA-AgNPs/CS gel with different molar ratios of CS:SA are shown in Figure 7e–g.

**Figure 8:**
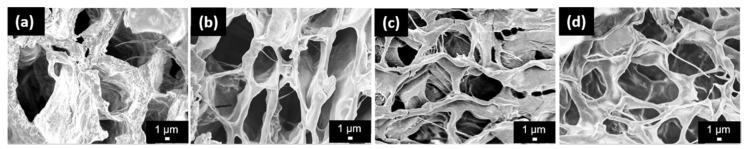
SEM images of (**a**) SA/CS (CS:SA = 0.75:1), (**b**) SA-AgNPs/CS (CS:SA = 0.5:1), (**c**) SA-AgNPs/CS (CS:A = 0.75:1), and (**d**) SA-AgNPs/CS (CS:SA = 1:1) gels.

As shown in the SEM micrographs, the pore size of the SA-AgNPs/CS gel was smaller than that of the SA/CS gel at all molar ratios of CS:SA (Figure 8). The average pore sizes of all samples are shown in Table 1. Additionally, the enhancement of the Young’s modulus of the SA-AgNPs could be attributed to the formation of uniform nucleation sites during the formation of SA-AgNP. According to Zhang et al., the mechanical properties of the gels can be improved by the inclusion of AgNP [47].

### 3.5. Water Resistance of the SA-AgNPs/CS Gels

**Figure 9:**
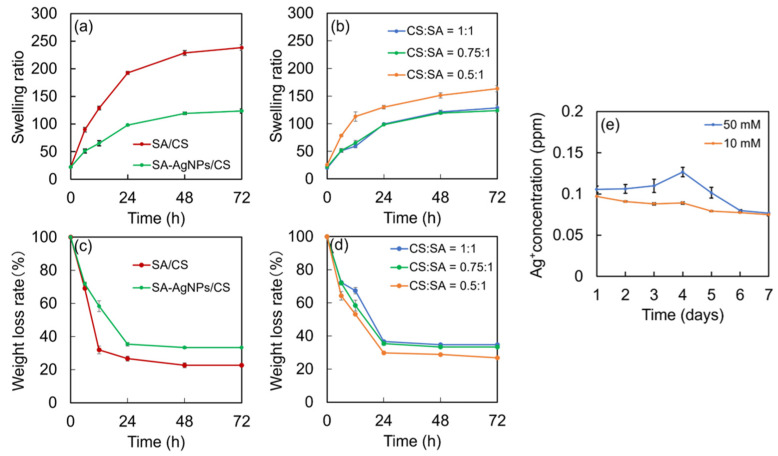
Swelling ratios of (**a**) the SA-AgNPs/CS and SA/CS gels, and (**b**) the SA-AgNPs/CS gel with different molar ratios of CS:SA. Residual weights of (**c**) the SA-AgNPs/CS and SA/CS gels (CS:SA = 0.75:1) and (**d**) the SA-AgNPs/CS gel with different molar ratios of CS:SA. (**e**) Concentration of Ag^+^ ions released from SA-AgNPs/CS gels fabricated using 50 and 10 mM AgNO_3_.

As shown in Figure 9a, the swelling ratios of the SA-AgNPs/CS and SA/CS gels increased with time. The SA-AgNPs/CS gel exhibited a lower swelling ratio than the SA/CS gel, possibly due to an increase in the crosslinking density due to the inclusion of SA-AgNPs, as described in Section 3.4. When the ratio of CS:SA was varied, the SA-AgNPs/CS gel with an CS:SA ratio of 0.75:1 exhibited the highest swelling ratio and crosslinking density (Figure 9b). Thus, the increase in crosslink density due to the inclusion of SA-AgNPs significantly influenced the swelling ratios of the gels. However, the weight loss values show the opposite trend: gels with a lower residual weight exhibited a larger swelling ratio, (Figure 9c,d). The low residual weights could be attributed to the utilization of acetic acid and GDL during fabrication of the gel. Figure 9e shows the release of Ag^+^ from the SA-AgNPs/CS gels synthesized using 10 and 50 mM of AgNO_3_. A release of less than 0.1 ppm of Ag^+^ for 7 days occurred when 10 mM of AgNO_3_ was used, whereas 50 mM resulted in a release of Ag^+^ in the range of 0.075–0.1 ppm. Studies on the effects of the release of AgNPs on cytotoxicity indicated that primary fibroblasts and hepatocytes isolated from mice survived at concentrations higher than 20 ppm of AgNPs [48]. Gradually released Ag^+^ entered the cells and interacted with the thiol groups of the enzymes and proteins. This affected their functions and protein synthesis, finally causing cell death. In this study, the SA-AgNPs/CS gels released less than 20 ppm Ag^+^, signifying low cytotoxicity. In addition, coating them with sugar chains enhanced the safety of the gels.

### 3.6. Antibacterial Activities

**Figure 10:**
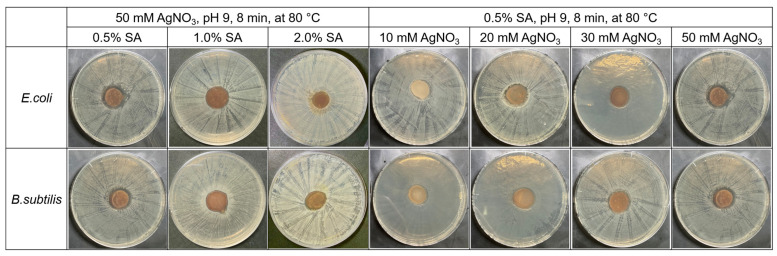
Inhabitation zones of the SA-AgNPs/CS gels obtained under several synthesis conditions.

**Figure 11:**
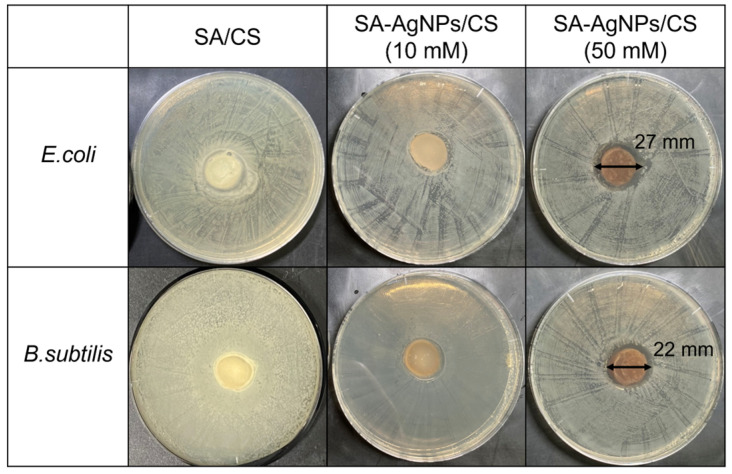
Inhibition zones of the SA/CS and SA-AgNPs/CS gels (fabricated with 10 and 50 mM AgNO_3_).

AgNPs exhibited antimicrobial effects against a variety of bacteria [49]. In this experiment, the antibacterial activity of the SA-AgNPs/CS gel was evaluated using the zone of inhibition method, with several conditions, as shown in Figure 10. All samples, except the sample obtained using 2% SA, showed an inhibition zone against *E. coli* and *B. subtilis*. Tokura et al. reported that the antimicrobial activity of a CS oligomer with a molecular weight of 9300 Da was likely caused by blocking the permeation of the nutrient through the cell wall of bacteria [50]. The SA/CS gel without AgNPs did not exhibit sufficient antibacterial activity (Figure 11), likely because the molecular weight of CS in this work was approximately 5.3 × 10^4^ Da, which was too large and could only block the nutrition supply through the cell. Unlike the SA/CS gel, the SA-AgNPs/CS gels showed significant antimicrobial ability against *E. coli* and *B. subtilis* because of the combination of the antimicrobial abilities of both AgNPs and chitosan. The SA-AgNPs/CS gel fabricated with 10 mM AgNO_3_ showed 17 and 16 mm inhibition zones against *E. coli* and *B. subtilis*, respectively, whereas the gel synthesized with 50 mM AgNO_3_ (a final SA-AgNP concentration of 20 ppm) showed 25 and 21 mm inhibition zones against *E. coli* and *B. subtilis*, respectively. The average value and the standard deviation were calculated from three measurements and are reported in Table 2. According to a report by Chen et al., PVA/SA/carboxymethyl chitosan (PVA/SA/CMCS) hydrogels containing SA-AgNPs and synthesized using 0.05% AgNO_3_ with 0.2% SA at 90 °C without microwave irradiation required a 12 h synthesis time. The PVA/SA/CMCS hydrogels containing SA-AgNPs showed 21 and 20 mm inhibition zones against *E. coli* and *S. aureus*, respectively [33]. Herein, the SA-AgNP/CS hydrogel containing SA-AgNPs and synthesized using microwave irradiation with an 8 min synthesis time showed high antimicrobial activity against *E. coli* and *B. subtilis*. It should be emphasized that the reaction time was reduced using our method.

### 3.7. Cytotoxicity

**Figure 12:**
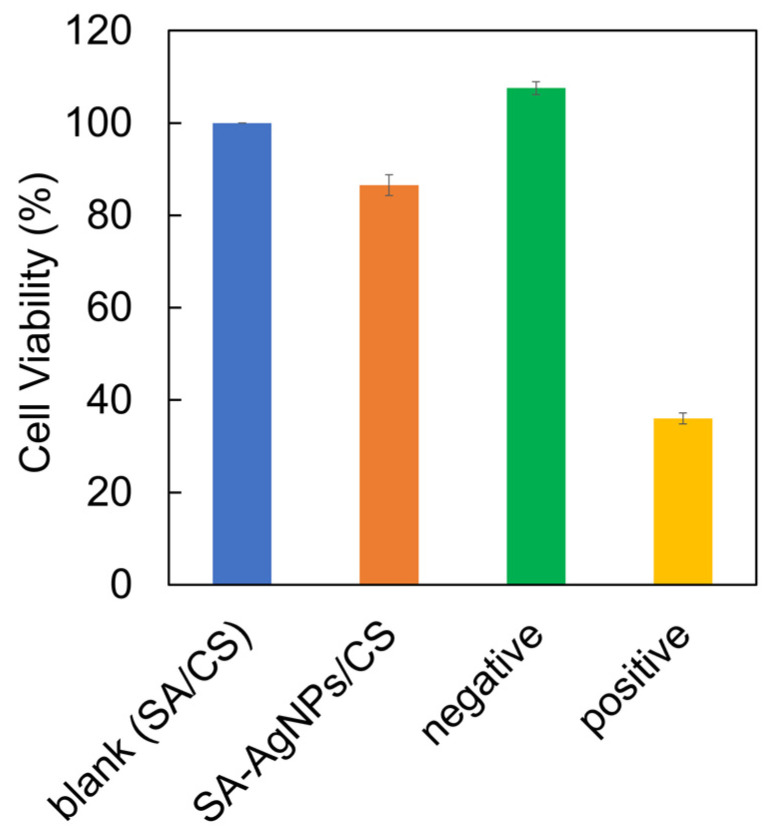
Cell viability of the SA-AgNPs/CS gel compared with the SA/CS gel (as a blank).

The FTIR spectra of the SA-AgNPs/CS gel before and after sterilization with EOG were used for the cytotoxicity experiment (Figure S1). The FTIR spectrum of the SA-AgNPs/CS gel did not change after sterilization. Chen et al. reported that PVA/SA/CMCS hydrogels containing SA-AgNPs synthesized using 0.05% AgNO_3_ with 0.2% SA at 90 °C for 12 h without microwave irradiation exhibited a cell survival rate of 80%, which was slightly lower than that without AgNPs, indicating low cell damage by the PVA/SA/CMCS/Ag hydrogels [33]. In this research, BALB-3T3 clone A31 cells (mouse fibroblasts) were used as the cell model to analyze the cytotoxicity of the SA-AgNPs/CS gel. Under the same conditions, the SA/CS gel extract was a blank, a HDPE film extract was a negative control, and a PU film with a 0.25% ZDBC extract was a positive control. The average value and the standard deviation were calculated from three measurements and are shown in Figure 12. The SA-AgNPs/CS gel with a final SA-AgNPs concentration of 20 ppm exhibited 87% cell viability, which was slightly lower than that of the blank (SA/gel). The cell survival rate was still high, especially when compared with that of the positive control. This finding was similar to that of other previous works [51]. Thus, it could be concluded that the SA-AgNPs have low toxicity towards living organisms at low concentrations.

## 4. Conclusions

In this study, SA was used as a stabilizer and a reducing agent for rapidly synthesizing SA-AgNPs from Ag^+^ using microwave irradiation, followed by the fabrication of SA-AgNPs/CS gels by mixing the SA-AgNPs with a basic CS solution. Usually, CS cannot be dissolved in a basic solution. In this work, CS (as a basic solution) was prepared using NaHCO_3_, and GDL was then added to the mixture of SA-AgNPs/CS with a low pH, specifically below the p*K*a of CS (~6.5). This resulted in the successful formation of a SA-AgNPs/CS gel that retained its shape. In addition, the gel with a CS:SA value of 0.75 exhibited the highest Young’s modulus of 6 kPa. The synthesized gels containing 20 ppm-SA-AgNPs showed good mechanical properties, high antibacterial activity against *E. coli* and *B. subtilis* with inhibition zones of 25 and 21 mm, and a low cytotoxicity of 87% cell viability. Thus, the SA-AgNPs/CS gels fabricated here, which had high antimicrobial activity and biocompatibility, could be utilized as wound dressings and for numerous other applications.

## Figures and Tables

**Table 1:** The average values of the pore size of the SA/CS gel and the SA-AgNPs/CS gels at different molar ratios of CS:SA.

Sample	Average Pore Size (10^−3^ mm^2^)
SA/CS gel (CS:SA = 0.75:1)	0.58 ± 0.05
SA-AgNPs/CS gels (CS:SA = 0.5:1)	0.55 ± 0.02
SA-AgNPs/CS gels (CS:SA = 0.75:1)	0.37 ± 0.02
SA-AgNPs/CS gels (CS:SA = 1:1)	0.41 ± 0.02

**Table 2:** Average values of the inhibition zones of the SA/CS and SA-AgNPs/CS gels (fabricated with 10 and 50 mM AgNO_3_).

Bacteria	SA/AgNPs/CS (10 mM)	SA/AgNPs/CS (50 mM)
*E. coli*	17 ± 1 mm	25 ± 2 mm
*B. subtilis*	16 ± 2 mm	21 ± 1 mm

## Data Availability

Data will be made available on request.

## Supplementary Materials

The following supporting information can be downloaded at https://www.mdpi.com/article/10.3390/jfb14040199/s1. Figure S1: FT-IR spectra of SA-AgNPs/CS gels before and after sterilization.
