# Supplementary material for: Microwave-Assisted Incorporation of AgNP into Chitosan–Alginate Hydrogels for Antimicrobial Applications
Source: J Funct Biomater. 2023 Apr 4;14(4):199. doi: 10.3390/jfb14040199 (PMC10141964; doi:10.3390/jfb14040199)
Supplement: Supplementary file 1 [file jfb-14-00199-s001.zip › jfb-2278470-supplementary.pdf]

Supplementary Information

Microwave-Assisted Incorporation of AgNP into Chitosan–Alginate Hydrogels for Antimicrobial Applications

Takuma Oe[1], Duangkamol Dechojarassri[1,2], Sachiro Kakinoki[1,2], Hideya Kawasaki[1,2], Tetsuya Furuike[1,2], Hiroshi Tamura[1,2]

[1]Faculty of Chemistry, Materials and Bioengineering, Kansai University

[2]ORDIST, Kansai University, 3–3–35 Yamate–cho, Suita, Osaka 564–8680, Japan

*Corresponding author: E-mail: tamura@kansai-u.ac.jp, Tel: +81 6 6368 0871

Supplementary information figure captions

Fig. S1† FT-IR spectra of SA-AgNPs/CS gels before and after sterilization.

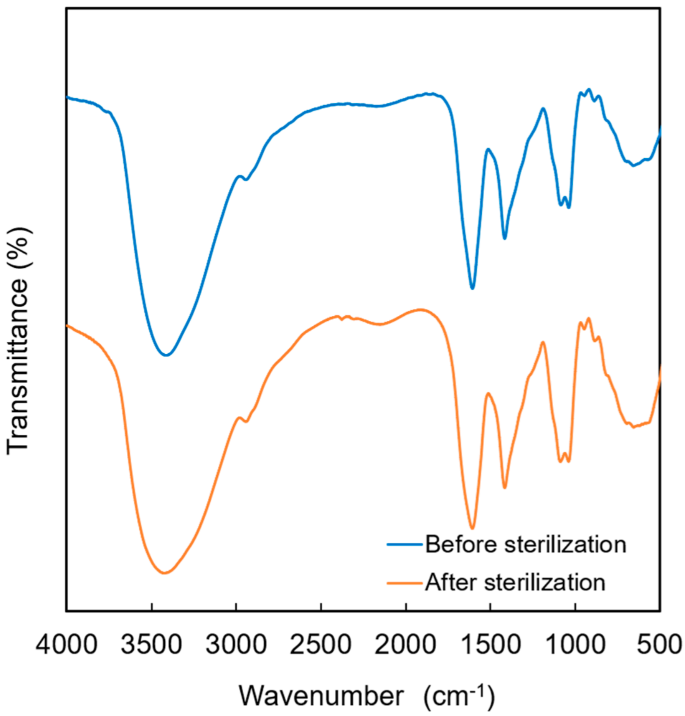

Fig. S1† FTIR spectra of SA-AgNPs/CS gels before and after sterilization.
